# Optimizing antiemetic treatment for chemotherapy-induced nausea and vomiting in Japan: Update summary of the 2015  Japan Society of Clinical Oncology Clinical Practice Guidelines for Antiemesis

**DOI:** 10.1007/s10147-020-01818-3

**Published:** 2020-11-08

**Authors:** Kenjiro Aogi, Hideki Takeuchi, Toshiaki Saeki, Keisuke Aiba, Kazuo Tamura, Keiko Iino, Chiyo K. Imamura, Kenji Okita, Yoshikazu Kagami, Ryuhei Tanaka, Kazuhiko Nakagawa, Hirofumi Fujii, Narikazu Boku, Makoto Wada, Tatsuo Akechi, Hirotoshi Iihara, Shoichiro Ohtani, Ayako Okuyama, Keiko Ozawa, Yong-Il Kim, Hidenori Sasaki, Yasuo Shima, Masayuki Takeda, Eijiro Nagasaki, Toshihiko Nishidate, Takahiro Higashi, Kouichi Hirata

**Affiliations:** 1 Department of Breast Oncology, National Hospital Organization Shikoku Cancer Center, Ehime, Japan; 2 Department of Breast Oncology, Saitama Medical University, Saitama, Japan; 3 Department of Breast Surgical Oncology, Japan Organization of Occupational Health and Safety Yokohama Rosai Hospital, Yokohama, Japan; 4 Department of Breast Oncology, Saitama Medical University International Medical Center, Saitama, Japan; 5 Division of Clinical Oncology/Hematology, Department of Internal Medicine, Jikei University School of Medicine, Tokyo, Japan; 6 Todachuo General Hospital, Saitama, Japan; 7 General Medical Research Center, Fukuoka University Hospital, Fukuoka, Japan; 8 Department of Adult Nursing, National College of Nursing, Tokyo, Japan; 9 Department of Clinical Pharmacokinetics and Pharmacodynamics, Keio University, Tokyo, Japan; 10 Advanced Cancer Translational Research Institute, Showa University, Tokyo, Japan; 11 Department of Surgery, Surgical Oncology and Science, Sapporo Medical University Postgraduate School of Medicine, Sapporo, Hokkaido Japan; 12 JR Sapporo Hospital, Sapporo, Hokkaido Japan; 13 Division of Radiation Oncology, Department of Radiology, Showa University School of Medicine, Tokyo, Japan; 14 Department of Pediatric Hematology and Oncology, Saitama Medical University International Medical Center, Saitama, Japan; 15 Department of Medical Oncology, Faculty of Medicine, Kindai University, Osaka, Japan; 16 Department of Clinical Oncology, Jichi Medical University, Tochigi, Japan; 17 Gastrointestinal Medical Oncology Division, National Cancer Center Hospital, Tokyo, Japan; 18 Department of Psycho-Oncology and Palliative Medicine, Osaka International Cancer Institute, Osaka, Japan; 19 Department of Psychiatry and Cognitive-Behavioral Medicine, Nagoya City University Graduate School of Medical Sciences, Aichi, Japan; 20 Department of Pharmacy, Gifu University Hospital, Gifu, Japan; 21 Department of Breast Surgery, Hiroshima City Hiroshima Citizens Hospital, Hiroshima, Japan; 22 Center for Cancer Control and Information Services, National Cancer Center, Tokyo, Japan; 23 Department of Nursing, NTT Medical Center Tokyo, Tokyo, Japan; 24 Department of Medical Oncology, Seirei Hamamatsu General Hospital, Sizuoka, Japan; 25 Department of Medical Oncology, Yodogawa Christian Hospital, Osaka, Japan; 26 Division of Medical Oncology, Hematology and Infectious Disease, Department of Medicine, Fukuoka University Hospital, Fukuoka, Japan; 27 Department of Palliative Medicine, Tsukuba Medical Center Hospital, Ibaraki, Japan

**Keywords:** Antiemesis, Chemotherapy-induced nausea and vomiting, Clinical practice guideline

## Abstract

Patients with cancer should appropriately receive antiemetic therapies against chemotherapy-induced nausea and vomiting (CINV). Antiemetic guidelines play an important role in managing CINV. Accordingly, the first Japanese antiemetic guideline published in 2010 by the Japan Society of Clinical Oncology (JSCO) has considerably aided Japanese medical staff in providing antiemetic therapies across chemotherapy clinics. With the yearly advancements in antiemetic therapies, the Japanese antiemetic guidelines require revisions according to published evidence regarding antiemetic management worldwide. A revised version of the first antiemetic guideline that considered several upcoming evidences had been published online in 2014 (version 1.2), in which several updated descriptions were included. The 2015 JSCO clinical practice guideline for antiemesis (version 2.0) (in Japanese) has addressed clinical antiemetic concerns and includes four major revisions regarding (1) changes in emetogenic risk categorization for anti-cancer agents, (2) olanzapine usage as an antiemetic drug, (3) the steroid-sparing method, and (4) adverse drug reactions of antiemetic agents. We herein present an English update summary for the 2015 JSCO clinical practice guideline for antiemesis (version 2.0).

## Introduction

Antiemetic therapies against chemotherapy-induced nausea and vomiting (CINV) should be developed and incorporated into cancer care protocols, while a framework for high-quality management should be widely distributed to cancer care providers [1–3]. Several international clinical guidelines that disseminate proper antiemetic treatment based on newly published evidences have been available [4–6]. For practical guidance in chemotherapy, the first Japanese Society of Clinical Oncology (JSCO) clinical practice guideline for antiemesis had been published in 2010 with its English version being published in 2016 [7]. Approximately 51.0% of medical staff throughout Japanese chemotherapy clinics perform antiemetic therapies according to this guideline, while 42.6% use this as a reference [8]. Thus, this guideline has considerably helped antiemesis treatment across Japanese clinics.

Some international antiemetic guidelines, such as those by the American Society of Clinical Oncology (ASCO), National Comprehensive Cancer Network (NCCN), and Multinational Association of Supportive Care in Cancer (MASCC), had been revised according to recently available evidence [4–6]. Consistent with this, the first JSCO antiemetic guideline had been revised and published online in 2014 as version 1.2, with further revisions thereto resulting in the publication of the JSCO clinical practice guideline for antiemesis version 2.0 in 2015 [9].  

## Methods & Results

Accordingly, the updated 2015 JSCO guideline contains four major revisions regarding (1) changes in emetogenic risk categorization for anticancer agents, (2) olanzapine usage as an antiemetic drug, (3) the steroid-sparing method, and (4) adverse drug reactions of antiemetic agents. Apart from the aforementioned revisions, the 2015 JSCO guideline discussed and provided updates on clinical antiemetic concerns. In addition, an online version of the said guideline (version 2.2) had been made available in 2018.

We herein present an English update summary of the 2015 JSCO clinical practice guideline for antiemesis (version 2.0).

## Materials and methods

A working group of the JSCO developed the first and second version of the clinical practice guideline for antiemesis based on the Appraisal of Guidelines for Research and Evaluation (AGREE) II instrument (https://www.agreetrust.org/resource-centre/agree-ii/), a widely used standard for assessing the methodological quality of practice guidelines.

A draft of the guideline was developed based on systematically reviewed evidences for clinical questions (CQs). However, domestic factors, including ethnicity and health policy formation at the system level, required further consideration. Hence, a consensus was reached by all medical practitioners attending a consensus meeting, during which recommendations for antiemetic treatments were discussed considering Japanese medical circumstances.

### Literature search strategy

The major international guidelines (i.e., NCCN, MASSC/ESMO, and ASCO) had been utilized as sources of information [4–6], similar to the first version. A systematic review and meta-analysis of the effectiveness of antiemetic therapies were performed using MEDLINE searches and the Cochrane library [10]. Available meeting abstracts from the ASCO and MASSC annual meetings were also reviewed.

### Conflicts of interest for the guideline

The update committee for the second version was assembled in accordance with ASCO's Conflict of Interest (COI) Management Procedures for Clinical Practice Guidelines (https://www.asco.org/guidelinescoi). Subsequently, the COI committee reviewed the COI of each member.

### Recommendation grade used in the guideline

Similar to the first version, recommendation grades were established as a guide for evidence evaluation:

A: Strongly recommended for clinical practice.

B: Recommend for clinical practice.

C1: Clinical practice may be useful despite the lack of high-level scientific evidence.

C2: Not recommended due to insufficient scientific evidence.

D: Clinical practice should be avoided.

### Emetogenic risks of intravenous and oral chemotherapeutic agents

Tables 1 and 2 discuss the emetogenic risk categorization for anticancer agents provided in the outline portion of the second version. Emetogenic risks of intravenous and oral chemotherapeutic agents presented in the aforementioned tables are based on recommendations established with a high level of consensus in several guidelines, such as those by the NCCN, MASSC, and ASCO, and have been modified according to clinical data in published literatures considering the medical circumstances in Japan.

**Table 1:** Emetogenic risk category for intravenous chemotherapeutic agents

JSCO emetogenic risk category	Agent (regimen)	
High emetogenic risk (emetic frequency: > 90%)	Cisplatin Cyclophosphamide (> 1500 mg/m^2^) Dacarbazine Doxorubicin + Cyclophosphamide Epirubicin + Cyclophosphamide	*Altretamine* *Carmustine (> 250 mg/m*^*2*^*)* *Mechlorethamine* *Streptozocin*
Moderate emetogenic risk (emetic frequency: 30–90%)	Interleukin-2 (> 12–15 million IU/m^2^) Busulfan (> 4 mg/day) Carboplatin Azacitidine Cyclophosphamide (≤ 1500 mg/m^2^) Cytarabine (> 200 mg/m^2^) Actinomycin D Bendamustine Clofarabine Daunorubicin Doxorubicin Epirubicin Idarubicin Ifosfamide Interferon α (≥ 10 million IU/m^2^) Irinotecan Melphalan (≥ 50 mg/m^2^)	Methotrexate (≥ 250 mg/m^2^) Oxaliplatin (≥ 75 mg/m^2^) Nedaplatin Enocitabine Therarubicin Amrubicin Arsenic trioxide Temozolomide *Amifostine (≥ 300 mg/m*^*2*^*)* *Carmustine (≤ 250 mg/m*^*2*^*)*
Low emetogenic risk (emetic frequency: 10–30%)	Interleukin-2 (≤ 12 million IU/m^2^) Brentuximab vedotin Cytarabine (100–200 mg/m^2^) Cabazitaxel Docetaxel Liposomal doxorubicin Etoposide Eribulin 5-Fluorouracil Gemcitabine Interferon-α (5–10 million IU/m^2^) Methotrexate (50–250 mg/m^2^) Mitomycin C	Mitoxantrone Nab-paclitaxel Paclitaxel Pemetrexed Trastuzumab emtansine Topotecan Pentostatin Nimustine Ranimustine *Amifostine (*< *300 mg)* *Carfilzomib* *Floxuridine* *Ixabepilone* *Omacetaxine* *Pralatrexate* *Romidepsin* *Ziv-aflibercept*
Minimal emetogenic risk (emetic frequency: < 10%)	l-Asparaginase Alemtuzumab Ipilimumab Interferon-α (≤ 5 million IU/m^2^) Ofatumumab Bevacizumab Bleomycin Bortezomib Cetuximab Cladribine Cytarabine (< 100 mg/m^2^) Fludarabine Gemtuzumab ozogamicin Methotrexate (≤ 50 mg/m^2^) Temsirolimus Trastuzumab Nivolumab Nelarabine Panitumumab	Peginterferon Pertuzumab Peplomycin Ramucirumab Rituximab Vinblastine Vincristine Vinorelbine Vindesine *Decitabine* *Denileukin diftitox* *Obinutuzumab* *Dexrazoxane* *Pegaspargase* *Pembrolizumab* *Siltuximab* *Valrubicin* *Liposomal vincristine*

*JSCO* Japan Society of Clinical Oncology

*Agents in Italics are not approved for clinical practice use in Japan

**Table 2:** Emetogenic risk category for oral chemotherapeutic agents

JSCO emetogenic risk category	Agent (regimen)	
High emetogenic risk (emetic frequency: > 90%)	Procarbazine *Hexamethylmelamine*	
Moderate emetogenic risk (emetic frequency: 30–90%)	Cyclophosphamide Temozolomide Trifluridine–tipiracil	Imatinib Crizotinib *Vinorelbine*
Low emetogenic risk (emetic frequency: 10–30%)	Alectinib Capecitabine Etoposide Everolimus Fludarabine Tegafur–Uracil (UFT) Thalidomide	S-1 Sunitinib Lapatinib Lenalidomide
Minimal emetogenic risk (emetic frequency: < 10%)	Erlotinib Gefitinib Hydroxyurea Melphalan	Methotrexate Sorafenib *Chlorambucil* *6-Thioguanine*

*JSCO* Japan Society of Clinical Oncology

*Agents in Italics are not approved for clinical practice use in Japan

Emetogenic risk is evaluated according to the percentage of patients who experience acute emesis within 24 h of initiation/administration of the anticancer agent and is categorized in the same manner as that in the first version.

High emetogenic risk: 90% or more patients experience acute emesis.

Moderate emetogenic risk: 30–90% of patients experience acute emesis.

Low emetogenic risk: 10–30% of patients experience acute emesis.

Minimal emetogenic risk: fewer than 10% of patients experience acute emesis.

## Results

### Summary of major updated issues

The second version of Japanese antiemetic guideline included the following major updated issues from the first version (Tables 3, 4):

1. Changes in emetogenic risk categorization for anticancer agents (Tables 1, 2).
2. Description regarding the usage of olanzapine (CQ2 and CQ3).
3. Description regarding steroid sparing (CQ2 and CQ3).
4. List of adverse toxicities for antiemetic agents (Table 5).

**Table 3:** Emetogenic risk category for radiation therapy

JSCO emetogenic risk category	Treated area	
High emetogenic risk (emetic frequency: > 90%)	Total body	
Moderate emetogenic risk (emetic frequency: 30–90%)	Upper abdomen	
Low emetogenic risk (emetic frequency: 10–30%)	Lower thorax Cranium (radiosurgery)	Pelvis Craniospinal
Minimal emetogenic risk (emetic frequency: < 10%)	Head and neck Cranium	Extremities Breast

No description changes were added in the second version

*JSCO* Japanese Society of Clinical Oncology

**Table 4:** Major updated issues in version 2

Major updated issues	
1	Changes in emetogenic risk categorization for anti-cancer agents (Tables 1, 2)
2	Description regarding olanzapine usage (described in CQ2 and CQ3)
3	Description regarding steroid sparin (described in CQ2 and CQ3)
4	Adverse drug reactions of antiemetic drugs (Table 5)

1. The JSCO antiemetic guideline committee provided revised tables on emetogenic risk categorization for intravenous and oral anti-cancer agents (Tables 1, 2,3), listing new chemotherapeutic agents, such as molecular or immunotherapeutic agents, according to emetogenicity as described in studies and drug interview forms.
2. Studies have shown that olanzapine, a multi-acting receptor-targeted antipsychotics (MARTA), was effective in controlling late-onset nausea and vomiting associated with high- and moderate-risk anti-cancer drugs [11–16], which has been described mainly in CQ2 and CQ3.
3. To reduce the adverse effects of steroids, an administration method that does not use steroids on day 2–3 of AC therapy (i.e., steroid-sparing) was used. Indeed, phase III studies have shown that steroid-sparing was not inferiority to conventional steroids use, with other reports also showing the effects of steroid-sparing [17–20] (CQ2 and CQ3).
4. To perform the proper antiemetic therapy using antiemetic agents, adverse effects of these agents should be taken into consideration to explain for patients by medical staffs. The adverse toxicities for antiemetic agents were listed in Table 5 for ‘at a glance’.

### Clinical questions and recommendations

The working group of the JSCO antiemetic guideline adopted clinical questions (CQs) as the guideline format similar to that in the first version wherein 21 CQs were described. In the second version, however, the number of CQs was reduced to 18. Moreover, the second version revised the title and context of CQ4 from the first version while adding three new CQs (i.e., CQ11, CQ12, and CQ13). When no changes in the description of the CQs from the first version were present, a short discussion indicating such was added as the last line of each CQ.

The following are the CQs included herein:

CQ1. How should oral chemotherapeutic agent-induced nausea and vomiting be managed?

CQ2. How should cancer chemotherapy-induced acute nausea and vomiting be prevented?

CQ3. How should delayed nausea and vomiting after cancer chemotherapy be prevented?

CQ4. How do we use a second-generation serotonin (5-HT_3_) receptor antagonist?

CQ5. Are corticosteroids recommended for preventing nausea and vomiting?

CQ6. How should breakthrough nausea and vomiting be managed?

CQ7. How should lowly and minimally emetogenic chemotherapy-induced acute nausea and vomiting be managed?

CQ8. How is nausea and vomiting managed for particular regimens, such as multiple daily administrations of cisplatin?

CQ9. How should anticipatory nausea and vomiting be managed?

CQ10. How are emetogenic risks categorized for radiation therapy?

CQ11. What factors are associated with nausea and vomiting?

CQ12. How are antiemetic treatment effects evaluated?

CQ13. How should CINV among patients staying at home be managed?

CQ14. How should CINV among pediatric patients with malignancies be managed?

CQ15. Can nausea be differentiated from anorexia, pyrosis, and dyspepsia?

CQ16. How are various forms of agents appropriately selected and used?

CQ17. Which antiemetic drugs produce pharmacokinetic interactions?

CQ18. How should opioid-induced nausea and vomiting be managed?

#### CQ1. How should oral chemotherapeutic agent-induced nausea and vomiting be managed?

Recommendation (Grade C1): According to clinical study protocols designed to assess the efficacy of supportive cotreatments, suspension and/or dose reduction of chemotherapeutic agents should be considered to control at most grade 2 nausea and vomiting.

Emetogenic risks of oral chemotherapeutic agents are presented in Table 5. In Japan, oral fluoropyrimidine-based regimens have been frequently used as an adjuvant treatment to tegafur-uracil/leucovorin and capecitabine for colorectal cancer, S-1 for gastric cancer, and tegafur-uracil for breast and lung cancers, with multiple clinical trials demonstrating reasonable efficacy. Moreover, Japanese clinical practice guidelines have indicated that S-1 and tegafur-uracil/leucovorin are effective agents for advanced gastric and colorectal cancers. Although these oral chemotherapeutic agents have lower emetogenicity when administered alone, adverse digestive events have been found to occur following repeated daily administration. Hence, antiemetic treatments are important to achieve higher drug adherence and optimize treatment effects.

The 2015 NCCN guidelines recommend the daily administration of metoclopramide, prochlorperazine, haloperidol, etc. (including lorazepam and H_2_ receptor antagonists if necessary) as oral agents, including drugs with moderate and minimal risk.

Randomized control studies showing the efficacy of these oral anticancer drugs generally provided antiemetic treatments to patients when grade 2 nausea/vomiting. When such cannot to be controlled by antiemetic treatments, suspending administration and/or dose reduction was commonly observed.

#### CQ2. How should cancer chemotherapy-induced acute nausea and vomiting be prevented?

Recommendation (Grade A): A triple regimen consisting of neurokinin 1 (NK_1_) receptor antagonist (aprepitant), serotonin (5-hydroxytryptamine: 5-HT_3_) receptor antagonist, and dexamethasone is recommended for acute emesis during highly emetogenic cancer chemotherapy.

Recommendation (Grade A): Regimens containing 5-HT_3_ receptor antagonists and dexamethasone are generally recommended for acute emesis during moderately emetic cancer chemotherapy. For certain chemotherapy regimens, the addition of NK_1_ receptor antagonists to 5-HT_3_ receptor antagonist and dexamethasone regimens have been considered.

**Fig. 1:**
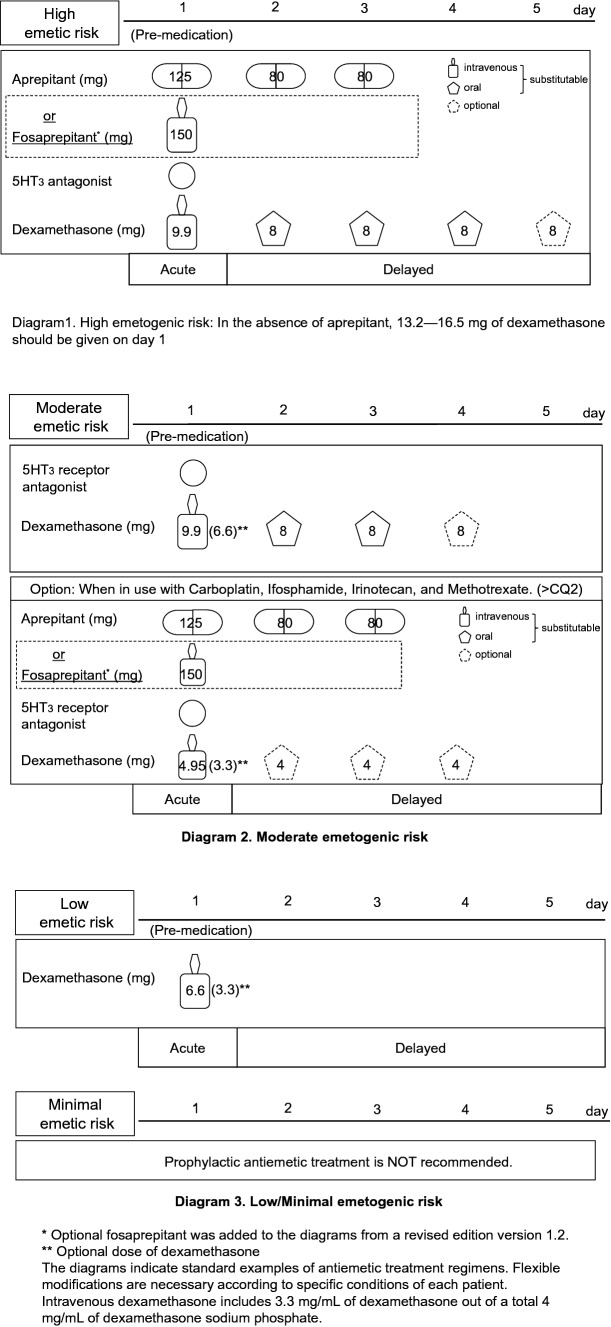
Diagram of antiemetic treatments for intravenous cancer chemotherapy

Acute onset nausea and vomiting occurs within a few minutes to several hours, with the intensity generally peaking within 5–6 h after chemotherapy administration and recovery usually taking place within 24 h. Considering that the unfavorable side effects of nausea and vomiting are associated with poor treatment adherence and effects, CINV management has been considered essential for successful cancer chemotherapy. In addition, incomplete prevention of acute emesis may lead to uncontrollable delayed emesis [21]. Hence, according to the four emetogenic risk categories indicated in CQ2 and CQ3, appropriate antiemetic treatments are needed upon initiating chemotherapy. The standard model for antiemetic treatment regimens is detailed in the four diagrams of Fig. 1. In the high emetogenic risk diagram, evidence for the antiemetic action of AC regimens, which was obtained from clinical trials of other high emetic cancer agents, suggested no additional effects of dexamethasone after day 2. Upon publication of the first guideline, oral aprepitant had been the only NK_1_ receptor antagonist available for clinical use in Japan. Subsequently, the Japanese Ministry of Health, Labour and Welfare had approved the use of fosaprepitant, an intravenous NK_1_ receptor antagonist, in November 2011. Accordingly, the diagram had been immediately modified to included additional information regarding fosaprepitant as a minor revision to the guideline.

(1) High emetogenic risk

Aprepitant (or fosaprepitant) in combination with a 5-HT_3_ receptor antagonist and dexamethasone is recommended for high-risk anticancer drugs as recommended by guidelines produced with a high level of consensus.

A triple combination consisting of oral NK_1_ receptor antagonist aprepitant (125 mg) or intravenous fosaprepitant (150 mg), a 5-HT_3_ receptor antagonist, and 12 mg of dexamethasone (injectable: 9.9 mg) is recommended. Studies have shown that the combined use of the aforementioned three agents with aprepitant promoted better antiemetic activity compared to the conventional combination of a 5-HT_3_ receptor antagonist and dexamethasone [22–24]. One study found that fosaprepitant was not inferiority to aprepitant in combination with ondansetron and dexamethasone for cisplatin therapy [25].

While palonosetron, a second-generation 5-HT_3_ receptor antagonist, had the same preventive effect on acute emesis as other drugs following direct comparisons between single agents and in combination with dexamethasone, it was found to be superior in the prevention of delayed emesis [26, 27] (CQ3). In two-drug combinations with a conventional 5-HT_3_ receptor antagonist, dexamethasone (CQ5) had been provided at a dose of 16–20 mg (injectable 13.2–16.5 mg). However, the area under the concentration–time curve (AUC) for dexamethasone increases when combined with aprepitant due to the latter’s influence on CYP3A4, thereby requiring a dose reduction to 12 mg (injectable 9.9 mg) for triple combinations. Although aprepitant is usually administered over 3 days, insufficient effects could prompt additional administration up to 5 days.

The 2015 NCCN guideline recommends the MARTA olanzapine (10 mg orally, days 1–4) instead of aprepitant in combination with palonosetron and dexamethasone. Indeed, the results of a phase III randomized controlled trial showed that olanzapine was equivalent to aprepitant in combination with palonosetron and dexamethasone for highly emetogenic anticancer drugs, such as cisplatin and AC agents [16]. However, the used of olanzapine in Japan requires considerable care due to adverse events, such as sleepiness and glucose intolerance. Other options, including lorazepam, H_2_ receptor antagonists, or a proton pump inhibitor may be additionally used depending on the situation (Fig. 1 and Diagram 1).

(2) Moderate emetogenic risk

For moderately risk anticancer drugs, a combination of 5-HT_3_ receptor antagonists and dexamethasone is recommended. For specific anticancer drug regimens, aprepitant can be added according to each patient’s condition.

Although a combination of 5-HT_3_ receptor antagonists and dexamethasone (8–12 mg; injectable: 6.6–9.9 mg) has generally been used, aprepitant (125 mg) has been recommended in combination with some anticancer drugs (carboplatin, ifosfamide, irinotecan, methotrexate, etc.). In such cases, the dose of dexamethasone is reduced to 4–6 mg (injectable drug: 3.3–4.95 mg) (Fig. 1 and Diagram 2).

A phase III multicenter randomized controlled trial in Japan including patients with colorectal cancer receiving oxaliplatin-based chemotherapy revealed that those who received aprepitant/fosaprepitant in combination with 5-HT_3_ receptor antagonists and dexamethasone had better nausea and vomiting control during the overall and delayed phase compared to those who received a combination of 5-HT_3_ receptor antagonists and dexamethasone [28].

(3) Low emetogenic risk (CQ7)

A single 4- to 8-mg dose of dexamethasone (injectable: 3.3–6.6 mg) is recommended for lowly emetogenic anticancer agents. Furthermore, depending on circumstances, prochlorperazine or metoclopramide can also be used (Fig. 1 and Diagram 3).

(4) Minimal emetogenic risk

Antiemetics have been deemed unnecessary for minimal risk anticancer agents (Figs. 1 and 3).

#### CQ3. How should delayed nausea and vomiting after cancer chemotherapy be prevented?

Recommendation (Grade A): A combined regimen consisting of NK_1_ receptor antagonist (aprepitant) and dexamethasone is recommended for treating delayed emesis during highly emetogenic cancer chemotherapy.

Recommendation (Grade A): A single administration of dexamethasone is basically recommended for delayed emesis during moderately emetic cancer chemotherapy. In some cases, regimens comprising NK_1_ antagonists and/or dexamethasone can be considered.

Delayed onset of nausea and vomiting has been shown to occur more than 24 h after chemotherapy administration. Under these circumstances, control of delayed emesis is essential for maintaining patients’ quality of life, motivation for further treatment, and mental health. In specific cases requiring dexamethasone restriction, 2–4 days of 5-HT_3_ antagonist is recommended instead of dexamethasone.

(1) High emetogenic risk

For delayed vomiting with highly emetogenic anticancer drugs, a combination of NK_1_ receptor antagonist aprepitant and dexamethasone is recommended.

Randomized controlled trials and pooled results have shown that a combination of 4–8 mg of oral dexamethasone (days 2–3) and 80 mg of oral aprepitant (days 2–3), an NK_1_ receptor antagonist, is better useful than dexamethasone alone [29–32]. Moreover, this two-drug combination can better suppress delayed emesis compared to a combination of 5-HT_3_ receptor antagonists and dexamethasone (21% vs. 36%; *p* < 0.001) [33].

Highly emetogenic regimens containing anthracycline anticancer drugs and cyclophosphamide differ from those utilized in clinical trials. Available evidence on AC therapy has yet to prove that dexamethasone had an effect on days 2 and 3 [17]. Furthermore, for the purpose of reducing side effects of steroids, overseas phase III trials have shown that steroid-sparing methods, which eliminate the use of steroids on days 2–3 of AC therapy, was not inferior to regular steroid use [17, 18, 20]. However, reports have also found that steroids are effective from days 2 to 3 [19]. As such, no consensus has yet been reached on steroid-sparing according to clinical trials in 2015 (CQ5).

### Commentary

Recent evidence from Japan in the form of a phase III study (TRIPLE trial) comparing the antiemetic effects of palonosetron, dexamethasone, and aprepitant in combination with granisetron, dexamethasone, and aprepitant for highly emetogenic anticancer drug administration revealed that the palonosetron group, although not the primary endpoint, significantly suppressed nausea and vomiting in the late phase [34].

Another Japanese phase III study (WJOG 6811 B trial) that compared granisetron and palonosetron in combination with dexamethasone/fosaprepitant for a regimen containing anthracycline and cyclophosphamide for breast cancer reported that the palonosetron group was significantly associated with nausea and vomiting in the delayed phase [35].

(2) Moderate emetogenic risk

For delayed emesis with moderately emetic anticancer drugs, dexamethasone is used alone. Depending on the case, a combination of aprepitant and dexamethasone or 5-HT_3_ receptor antagonists is used.

Studies have found that antiemetic regimens in combination with 5-HT_3_ receptor antagonists or dexamethasone provided no advantage compared to monotherapy [36, 37]. Hence, the cost-effectiveness of 5-HT_3_ receptor antagonists has remained controversial (palonosetron was not included in this review) [38].

However, in cases where dexamethasone cannot be used due to hepatitis, etc., 5-HT_3_ receptor antagonists may be used. Furthermore, results of a phase III study suggested that administration of palonosetron alone was sufficient for controlling delayed emesis [39]. Thus, the use of palonosetron alone can be one of the options for late emesis at present. Moreover, some phase III trials have shown that 5-HT_3_ receptor antagonists and corticosteroids have equivalent antiemetic and QOL improving effects [40].

The 2015 NCCN guideline and clinical trials have also indicated that aprepitant along or in combination with dexamethasone was efficacious for delayed emesis [1, 41].

(3) Low emetogenic risk/minimal emetogenic risk

Antiemetics are generally not recommended for mild and minimal risk anticancer drugs, with no randomized controlled trials having been conducted on the same (see Fig. 1 and Diagram 3).

#### CQ4. How do we use a second-generation serotonin (5-HT_3_) receptor antagonist?

Recommendation (Grade C1): Second-generation 5-HT_3_ receptor antagonists are preferred when used in the following context: NK_1_ receptor antagonist + 5-HT_3_ receptor antagonist (day 1) + dexamethasone (days 1–4) as prophylaxis for CINV during highly emetogenic chemotherapeutic regimens (except with cisplatin less than 50 mg/m^2^ and CHOP therapy).

Recommendation (Grade C1): As prophylaxis for CINV during MEC, especially when using a relatively highly emetogenic anticancer drugs, first-generation 5-HT_3_ receptor antagonists are recommended when NK_1_ receptor antagonists are used. In the absence of NK_1_ receptor antagonists, second-generation 5-HT_3_ receptor antagonists are preferred.

Several 5-HT_3_ receptor antagonists have currently been available in Japan, with their efficacy in managing CINV having been demonstrated, particularly under conditions of acute-phase emesis. However, the efficacy of such agents in the treatment of delayed emesis has remained controversial given the absence of further antiemetic effects of additional treatments after the initial occupation of the 5-HT_3_ receptors with antagonistic agents.

The TRIPLE trial, which compared the antiemetic effects of a palonosetron, dexamethasone, aprepitant arm with a granisetron, dexamethasone, and aprepitant arm for cisplatin-containing highly emetogenic chemotherapeutic regimen, showed that the palonosetron arm significantly suppressed delayed nausea and vomiting, though not the primary endpoint [34]. For less than 50 mg/m^2^ of cisplatin and CHOP regimen, evidence has shown that second-generation 5-HT_3_ receptor antagonists were no superior to first-generation ones.

When using an AC regimen without NK_1_ receptor antagonists, palonosetron was proven to be not inferior to granisetron during the acute phase but superior to granisetron during the delayed phase [27].

The SENRI trial [28] revealed that triple antiemetic therapy comprising a NK_1_ receptor antagonist, 5-HT_3_ receptor antagonist, and dexamethasone was superior to double therapy comprising a 5-HT_3_ receptor antagonist and dexamethasone in suppressing vomiting rates during oxaliplatin-based MEC regimen.

Furthermore, triple antiemetic therapy had been found to be superior to double therapy comprising a 5-HT_3_ receptor antagonist and dexamethasone in a subset analysis of a randomized study for antiemetic therapy using a MEC regimen [41].

No conclusive results have been available to elucidate the difference in efficacy between first- and second-generation NK_1_ receptor antagonists during MEC regimens.

#### CQ5. Are corticosteroids recommended for preventing nausea and vomiting?

Recommendation (Grade A): Corticosteroids is an effective antiemetic agent at recommended doses, which are determined according to the emetogenic risk categories of chemotherapeutic regimens.

Although corticosteroids have been used as prophylaxis against emesis during cancer chemotherapy for 25 years [42], their mechanism of action remains unclear compared to those of 5-HT_3_ and NK_1_ antagonists, which have recently been approved with clear details regarding their mechanisms. Although multiple classes of corticosteroid are available, dexamethasone and methylprednisolone have been most frequently used antiemetics, with strong evidence supporting their effects [43, 44]. However, the efficacy of high dose dexamethasone has yet to be compared with 20-mg treatments in either Western [43, 44] or Japanese populations [45].

#### CQ6. How should breakthrough nausea and vomiting be managed?

Recommendation (Grade B): Fixed, around-the-clock administration of various drugs should be considered according to the patient symptoms. In addition, antiemetic 5-HT_3_ receptor antagonists should be replaced with another drug of the same type.

Breakthrough nausea and vomiting are defined as the continuous onset of nausea and vomiting even after prophylactic administration of antiemetics.

A systematic review of antiemetic treatments in patients with advanced cancer showed that metoclopramide was superior to placebo and equivalent to ondansetron, although response rates were only 23–36% for nausea and 18–52% for vomiting, respectively [46]. Moreover, a randomized clinical trial including patients with advanced cancer showed that additional dexamethasone for nausea following the failure of antiemetic responses to metoclopramide had no significant effects [47].

#### CQ7. How should lowly and minimally emetogenic chemotherapy-induced acute nausea and vomiting be managed?

Recommendation (Grade B): During lowly emetogenic chemotherapy, dexamethasone should be considered according to the chemotherapeutic regimen and patient background.

Recommendation (Grade C1): Routine usage of dexamethasone is not recommended for minimally emetogenic chemotherapy.

Prophylactic antiemetic treatment is not recommended for lowly or minimally emetogenic chemotherapy. Nonetheless, some patients suffer from emesis during treatment with lowly/minimally emetogenic chemotherapies, necessitating flexible and appropriate treatment despite the absence of high-level evidence. The ASCO and MASCC/ESMO antiemetic guidelines have recommended the administration of 4–8 mg of dexamethasone [5, 6] and inclusion of prochlorperazine [48] and metoclopramide as optional antiemetics.

No descriptions were changed in the second version.

#### CQ8. How is nausea and vomiting managed for particular regimens, such as multiple daily administrations of cisplatin?

Recommendation (Grade B): A triple antiemetic regimen comprising 5-HT_3_ antagonists, dexamethasone, and aprepitant is recommended for preventing acute nausea and vomiting during cisplatin-containing chemotherapeutic regimens. Meanwhile, a double regimen comprising dexamethasone and aprepitant is recommended for delayed nausea and vomiting, even during regimens involving multiple daily cisplatin administrations.

Cisplatin, which has been widely accepted as a highly emetogenic chemotherapeutic agent, is commonly administered every 3 or 4 weeks at ≥ 50 mg/m^2^ for the treatment of various malignancies. However, differing cisplatin regimens have been established with reasonable supporting evidence, including multiple daily cisplatin doses at < 50 mg/m^2^ for cholangiocarcinomas, bladder cancer, and germinomas [49, 50] and continuous cisplatin injections at 100 mg/m^2^ over 4 days for non-Hodgkin lymphomas.

No descriptions were changed in the second version.

#### CQ9. How should anticipatory nausea and vomiting be managed?

Recommendation (Grade B): Initially, complete prevention of emesis is essential during the acute and delayed phases so that patients never experience nausea and vomiting.

Recommendation (Grade B): Benzodiazepine is effective for anticipatory nausea and vomiting.

Recommendation (Grade B): Psychological therapies, such as systematic desensitization/behavioral treatment, relaxation therapy, and hypnotherapy for pediatric patients, effectively ameliorate anticipatory nausea and vomiting.

Anticipatory nausea and vomiting have been found to occur immediately prior to treatment and reflects previous negative experiences of cancer chemotherapy [51–53], although nausea is more common than vomiting among such cases. The ideal prophylaxis for this symptom is to perform complete emesis prevention upon commencement of treatment [52–55].

No descriptions were changed in the second version.

#### CQ10. How are emetogenic risks categorized for radiation therapy?

Recommendation (Grade A): Emetogenic risks of radiation therapy are classified according to tissue targets and volumes for irradiation.

The risk of radiation-induced nausea and vomiting is categorized according to the percentage of patients with emesis. Moreover, the whole body and upper abdominal radiation therapy are likely to cause greater emesis, with the frequency of nausea and vomiting increasing with larger total doses and target tissue volumes [56, 57].

No descriptions were changed in the second version.

#### CQ11. What factors are associated with nausea and vomiting?

Recommendation (Grade C1): Treatment and patient factors influence the emetogenic risks of CINV. Treatment factors include emetogenicity and dosages of chemotherapeutic agents, tissue target, and radiation therapy volume, while relevant patient factors include age, gender, and alcohol consumption.

Patient factors, such as age [58], gender [58, 59], alcohol consumption [60], and experience of nausea gravidarum, have been shown to influence the emetogenicity of CINV. The 2015 NCCN guideline also shows that bowel obstruction, vestibulopathy, brain metastasis, electrolyte abnormality, uremia, opioid use, gastric atony, and mental disorders are potential risk factors for emesis. Treatment-related factors are classified into risk categories, with the antiemetics recommended for each category being fixed. However, no current consensus exists on how to deal with patient-related factors.

The CINV Study Group of Japan performed a survey evaluating the incidence of acute and delayed nausea/vomiting caused by highly and moderately emetogenic anticancer drugs [61]. In this survey, gender (female) and age (young) were identified as factors influencing CINV during the acute phase, while gender (female) was identified as a factor during the delayed phase.

#### CQ12. How are antiemetic treatment effects evaluated?

Recommendation (Grade A): Antiemetic treatment effects should be assessed at every visit for outpatients and within 24 h after administration of chemotherapy for admitted patients.

Recommendation (Grade C1): Strict assessments require patients to report their conditions to medical staff using self-reporting systems.

Recommendation (Grade C1): Continuous assessments will be performed from baseline (before treatment) and throughout the course of treatment for appropriate palliation of CINV.

No clear consensus or evidence has been available regarding the evaluation of nausea and vomiting among patients with cancer, as well as antiemetic use. Identifying the cause of emesis through clinical evaluation is important (CQ14 and CQ16). The common terminology criteria for adverse events (CTCAE) may be useful in evaluating side effects during chemotherapy, which are based on objective assessments by medical staff rather than subjective assessments by patients.

Applicable patient-directed subjective evaluations include the numerical rating scale (NRS), visual analog scale (VAS), verbal rating scale, and the Wong–Baker Face Rating Scale [62]. Moreover, the Index of Nausea, Vomiting and Retching [63], MASCC Antiemesis Tool [64], Morrow Assessment of Nausea And Emesis [65], and Functional Living Index-Emesis scores [66] may also be utilized as tools for evaluating changes in emesis and ensuing influences on quality of life.

#### CQ13. How should CINV among patients staying at home be managed?

Recommendation (Grade C1): Despite the lack of recommended treatments, management of nausea and vomiting during home treatment is important. Hence, treatment based on evidence is performed.

For outpatients, managing nausea/vomiting at home, which may be out of the medical staff’s reach, is an important issue directly linked to patients' QOL. Some reports have suggested that patients want to prioritize the control of delayed-phase nausea in practice [67, 68]. Accordingly, Tamura et al. reported that Japanese patients with cancer receiving chemotherapy were more concerned regarding delayed CINV than acute CINV [61]. However, medical staff have no definitive methods for evaluating CINV among patients staying at home after chemotherapy. No clinical trials regarding the effective treatment for delayed CINV, especially focusing on nausea and vomiting at home, had been identified. Hence, further studies concerning CINV at home are needed.

#### CQ14. How should CINV be managed among pediatric patients with malignancies?

Recommendation (Grade C1): Multidisciplinary management using 5-HT_3_ receptor antagonists, corticosteroids, and other antiemetic agents can control CINV, even among pediatric patients.

More than 70% of cancer among children are currently curable with modern and intensive therapeutic modalities, including high-dose chemotherapy with or without allogeneic stem cell transplantation. However, only a few reports have presented high-level evidence regarding antiemetic treatment among pediatric patients from Western populations [69–71]. Accordingly, such patients receive modified dosages based on the results of clinical trials among adult patients. Proper antiemetic treatments may allow pediatric patients to receive cancer chemotherapy without a decline in QOL.

#### CQ15. Can nausea be differentiated from anorexia, pyrosis, and dyspepsia? Which diseases produce symptoms of nausea and vomiting?

Recommendation (Grade B): No definitive evidence allows for the differentiation between nausea and anorexia, pyrosis, and dyspepsia. However, proton pump inhibitors (PPI) and H_2_ blockers are recommended for patients with these symptoms.

Recommendation (Grade C1): Antiemetic agents should be used according to accurate assessments of patient condition.

Symptoms of anorexia, pyrosis, and dyspepsia are caused by multiple factors related to digestive dysfunction and are frequently accompanied by nausea and other symptoms. Therefore, chemotherapy-induced nausea has not been strictly differentiated from other symptoms of digestive dysfunction. Nonetheless, PPI and H_2_ blockers are recommended as optional treatments for these symptoms [72].

In addition to treatments for CINV, patients with malignancies may suffer from nausea and vomiting due to the following conditions:

Partial or complete bowel obstruction.

Vestibulopathy.

Brain metastasis.

Electrolyte abnormality (hypercalcemia, hyponatremia, and hyperglycemia).

Uremia.

Other combinations of drugs, including opioids.

Gastric atony.

Anticipatory nausea and vomiting.

No descriptions were changed in the second version.

#### CQ16. How are various forms of agents appropriately selected and used?

Recommendation (Grade B): Patients should self-manage the use of oral agents. Considering that nausea and vomiting prevent patients from taking oral treatments, optional intravenous administration should be considered.

A meta-analysis of randomized control trials showed that oral and intravenous 5-HT_3_ receptor antagonists had similar effects [73]. Oral agents may have superior cost-effectiveness and convenience compared to intravenous agents, particularly when administered as tablets that disintegrate orally. On the other hand, intravenous agents may improve treatment adherence among pediatric patients.

#### CQ17. Which antiemetic drugs produce pharmacokinetic interactions?

Recommendation (Grade B): Aprepitant/fosaprepitant should be used carefully to avoid interactions with co-administered drugs, including certain chemotherapeutic agents.

Aprepitant acts as a substrate that induces and inhibits cytochrome P450 enzymes 3A4 (CYP3A4) and 2C9 (CYP2C9). Hence, aprepitant can alter plasma concentrations of co-administered drugs by interacting with these critical drug-metabolizing enzymes [74]. Chemotherapeutic agents that are metabolized by CYP3A4 include docetaxel, paclitaxel, etoposide, irinotecan, ifosfamide, imatinib, vinorelbine, vinblastine, and vincristine. Although doses for several chemotherapeutic agents used concurrently with aprepitant in phase III trials were not adjusted, such drugs should be used with caution [75, 76] given that aprepitant interacts with several non-chemotherapeutic drugs, including warfarin, dexamethasone, and methylprednisolonre [77]. Concurrent use of aprepitant temporarily reduces prothrombin time–international normalized ratio among patients receiving regimens containing warfarin, necessitating anticoagulant monitoring among these patients [29]. Aprepitant also increases the AUCs of corticosteroids dexamethasone, and methylprednisolone, necessitating appropriate reductions in corticosteroid doses (CQ7) [74]. However, to ensure anticancer effects, corticosteroid doses in chemotherapeutic regimens for malignant lymphoma should not be reduced, despite concomitant use of aprepitant.

No descriptions were changed in the second version.

#### CQ18. How should opioid-induced nausea and vomiting be managed?

Recommendation (Grade C1): Prophylactic antiemetic treatments using dopamine receptor antagonists for approximately 7 days during opioid therapy may be useful despite the lack of high-level evidence of efficacy and safety.

The World Health Organization ladder strongly recommends opioid use for cancer pain according to high-level evidences of efficacy and safety. Among the three opioid receptors, the μ and κ receptors are categorized as emetogenic, while the δ receptor exhibits antiemetic functions.

Patients frequently suffer from constipation, sleepiness, nausea, and vomiting upon initiation of opioid therapy. However, antiemetic treatments for opioid-induced emesis comprising dopamine receptor antagonist for 7 days (considering the side effects of dopamine receptor antagonists) may be important for successful pain control among patients with cancer, despite the lack of high-level evidence of efficacy and safety. Moreover, the differential diagnosis of other causes is important for patients suffering from emesis after opioid treatments.

### Adverse drug reactions of antiemetic agents

Adverse drug reactions of antiemetic agents have been considered obstacles hindering appropriate antiemetic therapies among patients with cancer. The update committee for the second version considered that information related to the adverse reaction of antiemetic drugs should be made available to patients with cancer, a list of which is presented in Table 5.

**Table 5:** Adverse drug reactions of the antiemetic agents

5-HT_3_ receptor antagonists Azasetron, Indisetron, Ondansetron, Granisetron, Tropisetron, Ramosetron, Palonosetron		
Main adverse drug reaction	Mental nervous system	Headache
	Digestive organ	Constipation
Infrequent but severe reaction	Immune system	Shock, anaphylaxis
	Circulatory organ	QT prolongation
NK_1_ receptor antagonists: Aprepitant, fosaprepitant		
Main adverse drug reaction	Mental nervous system	Headache
	Digestive organ	Constipation
	Respiratory	Hiccups
	Injection site (fosaprepitant)	Injection site pain
Infrequent but severe reaction	Skin	Stevens-Johnson syndrome
	Immune system	Anaphylaxis
Dexamethasone		
Main adverse drug reaction	Immune system	Induced infection, exacerbation of infection
	Mental nervous system	Depression, euphoria
Infrequent but severe reaction	Metabolism	Hyperglycemia
Phenothiazines: Metoclopramide, Prochlorperazine		
Infrequent but severe reaction	Mental nervous system	Latent dyskinesia
		Malignant syndrome
Benzodiazepines: Lorazepam		
Main adverse drug reaction	Mental nervous system	Sleepiness, dizziness
H_2_ receptor antagonists: Cimetidine, Nizatidine, Famotidine, Ranitidine, Lafutidine, Loxatidine		
Main adverse drug reaction	Mental nervous system	Headache
	Digestive organ	Constipation, diarrhea
Infrequent but severe reaction	Skin	Stevens-Johnson syndrome
	Immune system	Shock, anaphylaxis
Proton pump inhibitors: Esomeprazole, Omeprazole, Rabeprazole, Lansoprazole		
Main adverse drug reaction	Mental nervous system	Headache
	Digestive organ	Diarrhea/loose stools, abdominal pain
Infrequent but severe reaction	Skin	Stevens-Johnson syndrome, toxic epidermal necrolysis (TEN)
	Immune system	Shock, anaphylaxis
	Genitourinary system	Interstitial nephritis

## Discussion

Excellent and up-to-date cancer medical practitioners select appropriate medications based on the optimal course of treatment and safely maintain the therapeutic intensity while minimizing pain and sequelae leading to the maximum effect. With the establishment of treatment guidelines for various cancers in recent years, appropriate drug therapy selection has increased, and drug treatment regimens can be registered and managed in each facility. CINV can be representative of a patient’s pain even when the mechanisms of Emesis have been elucidated and drugs acting on it have been developed.

The JSCO had published their first clinical practice guideline for antiemesis in 2010, which had attracted considerable attention from medical professionals in Japan, including physicians, nurses and pharmacists. As previously described [8], the JSCO conducted an interview searching for the penetration of the antiemetic guideline among organizations participating in the 2012 JSCO annual meeting. In this survey, 586 (51.0%) Japanese medical staff provided antiemetic therapies according to the guideline, while 489 (42.6%) actually referred to it. After determining the discrepancy between guideline recommendations and medical practice, the study identified institutional clinical situation, domestic insurance application, and patient and doctor preferences as reasons for such a discrepancy [78].

To improve the quality of the antiemetic guideline, the JSCO organized a guideline evaluation team that functioned independently from the guideline update committee. A second version had been published based on the need for (1) creating guidelines considering the needs and current situation in Japan and (2) actively obtaining high-level evidence test results in Japan (https://www.jsco-cpg.jp/item/29/index.html). In addition, the Japanese antiemetic guideline has been updated every 2–3 years with the availability of new evidence, such as the addition olanzapine to standard therapy and steroid-sparing therapy. Carboplatin has been categorized as a moderately emetogenic agent by this second version guideline. However, the JSCO antiemetic guideline update committee recategorized carboplatin as a highly emetogenic chemotherapeutic agent according to clinical handling in Japan and guidelines with a high-level consensus. As such, the description for the categorization of carboplatin in the JSCO antiemetic guideline website and subsequent revised versions should be changed.

While the second version of the guideline had not been constructed based on the international guideline construction method introduced by Medical Information Network Distribution Service (Minds) 2014, the JSCO antiemetic guideline update committee will be following this methodology for the next version. For effective antiemetic care of Japanese patients with cancer, continuous improvement in the quality of the antiemetic guideline is imperative.

We herein presented an updated summary of the second version of the JSCO antiemetic guideline, which showed high concordance with other international antiemetic guidelines based on high-level evidence. Overall, proper application of antiemetic therapy may lead to excellent anticancer therapy outcome among Japanese patients with cancer.
